# Effects of Temperature and Packaging Atmosphere on Shelf Life, Biochemical, and Sensory Attributes of Glasswort (*Salicornia europaea* L.) Grown Hydroponically at Different Salinity Levels

**DOI:** 10.3390/foods13203260

**Published:** 2024-10-13

**Authors:** Chiara Sanmartin, Isabella Taglieri, Alessandro Bianchi, Prangthip Parichanon, Martina Puccinelli, Alberto Pardossi, Francesca Venturi

**Affiliations:** 1 Department of Agriculture, Food and Environment, University of Pisa, Via del Borghetto 80, 56124 Pisa, Italy; chiara.sanmartin@unipi.it (C.S.); isabella.taglieri@unipi.it (I.T.); prangthip.wu@gmail.com (P.P.); martina.puccinelli@agr.unipi.it (M.P.); alberto.pardossi@unipi.it (A.P.); francesca.venturi@unipi.it (F.V.); 2 Interdepartmental Research Centre “Nutraceuticals and Food for Health”, University of Pisa, Via del Borghetto 80, 56124 Pisa, Italy

**Keywords:** halophytes, modeling food shelf life, modified atmosphere packaging, phytochemical compositions, sea asparagus, sensory quality, storage conditions

## Abstract

Halophytes, such as *Salicornia* species, are promising new foods and are consumed for their pleasant salty taste and nutritional value. Since *Salicornia* is perishable, modified atmospheric packaging (MAP) can be a useful tool, in combination with proper temperature, to halt further quality degradation in this type of product. The purpose of this study was to investigate the effect of MAP, with or without refrigeration, to extend the shelf life of glasswort (*Salicornia europaea* L.) grown hydroponically (floating raft system) in a greenhouse with a nutrient solution containing 0 g/L (C) or 12.5 g/L of NaCl (T). The dry matter content, weight loss, respiration rate, biochemical composition, color, antioxidant capacity, and sensorial attributes were determined in shoots after harvest and during storage in plastic bags filled with technical air or with MAP at 4 or 20 °C for 120 h. At harvest, plants supplied with salt-enriched solution (T) showed a significant improvement in nutritional value and sensory profile. Storage in air at room temperature (20 °C) accelerated weight loss and diminished color stability, particularly in non-salinity samples (C), while MAP extended the shelf life of all the samples regardless of the storage temperature adopted. Optimal storage conditions were observed when MAP was combined with refrigeration, which allowed to effectively preserve shoots sensory acceptability for a period of about seven days. Future research could further explore the long-term effects on the nutritional value and sensory quality of *S. europaea* under various combinations of MAP and different storage temperatures ranging between 4 °C and 20 °C.

## 1. Introduction

Global warming affects weather patterns, resulting in heat waves, excessive rainfall, and droughts. Thus, global food insecurity has been increasing largely consequently. Moreover, it is expected that drought and salinity will spread worldwide, reducing plant growth and productivity in a wide variety of crop species. Furthermore, scientists are facing the challenging task of producing 70% more food to feed an additional 2.3 billion people by 2050 [1,2]. To adequately meet such food requirements of the next generation, it is crucial to produce crops with higher yields under drought and salt stress [3]. Agriculture must meet the demands of current and future generations while ensuring profitability, environmental health, and social and economic equality in order to be sustainable [4]. Consequently, sustainable agricultural practices are one of the most significant ways to promote food security and a healthy lifestyle, as agricultural activities produce nutritious food and provide a good sense of taste for consumers [5]. In addition, the increasing salinization of both soils and water resources, mainly due to anthropic activity, has promoted a new interest in salt-tolerant plants as potential crops for saline environments [6,7,8,9].

Halophytes can grow and reproduce in highly diverse saline habitats and conditions by evolving different adaptive mechanisms [10,11,12]. Therefore, saline agriculture with halophyte species could overcome the problems related to the depletion of freshwater resources and the expansion of areas with salinized soil [3,13]. Researchers have identified *Salicornia* spp. as the most promising halophytes in the Mediterranean area, and Europeans have historically used them as a folk medicine to cure a wide range of diseases due to its high content of minerals, fibers, and high-quality lipids and proteins [14,15,16]. Several *Salicornia* spp. are widespread in Italy, covering almost the country’s coasts [17]. One of these species is *S. europaea* L. (Syn.: *S. perennis* Willd. subsp. *perennans*; *S. patula* Duval-Jouve), which is consumed for its pleasant salty taste and nutritional value [18]. According to Lu et al. (2010) [19], subtropical deserts can support the growth of these annual, leafless, succulent halophytes. Several researchers focused on an increasingly grown *Salicornia* spp. in open fields and greenhouses for edible purposes [3,12,20]. Indeed, the glasswort is currently regarded as a high-value gourmet vegetable [17,21]. *Salicornia* spp. also showed great adaptability to soilless cultivation (or hydroponics). Different soilless growing systems, including the floating raft system [22,23], the nutrient film technique (NFT) [24], and substrate culture [20], have been tested for greenhouse production of fresh shoots of *Salicornia* spp. The floating raft system is broadly used for producing green vegetables with a short growing cycle at high plant density. Among the main advantages of soilless cultivation are the fast crop growth rate as a result of optimal water and mineral nutrient supply and the cleaning of leaves and shoots, which can thus be processed more easily in the case of ready-to-eat products [20,23,25,26]. In general, greenhouse soilless cultivation can allow year-round production of high-quality products with an efficient use of water and fertilizers [27].

Cold storage is a popular technique to maintain the quality of fresh products after harvest, but some issues related to energy costs and sustainability should be considered. Moreover, modified atmospheric packaging (MAP) can be a useful tool, alone or in combination with cold storage, to halt further quality degradation in different types of products [28,29,30]. MAP is nowadays the most effective method of food preservation and protection to extend the shelf-life of fresh fruits and vegetables [31,32]. This technique was generally applied to products with a high respiration rate, such as cut flowers [33] or flower vegetables, such as broccoli [34]. This technology works by preventing food from encountering oxygen on the surface, thus reducing oxidative decay and extending its shelf life. It also minimizes the need for chemical food preservatives, ensuring that the food remains authentic and fresh without altering its organoleptic qualities [35,36]. *Salicornia* shoots are highly perishable, as they were found to have a shelf life of only about 6 days at ambient temperature [19,28]. Furthermore, in the past years, different studies [19,37,38,39,40,41,42,43] have focused on the utilization and nutritional properties of halophytes or on the best storage conditions. Almost all these studies were conducted with plants collected in nature, and to the best of our knowledge, no study has been carried out on *Salicornia europaea* to investigate during storage the effect of MAP on the shelf-life and sensorial attributes of *S. europaea* cultivated in hydroponic systems.

In this context, the aim of this research project was to evaluate the feasibility of storing fresh shoots of *S. europaea*, grown in hydroponic systems under different saline conditions, using MAP to extend its shelf life, with or without refrigeration. A control sample, cultivated and stored under the same experimental conditions but packaged with technical air, was used for comparison. The study focused on the evaluation of biochemical compositions, antioxidant activity, color, respiratory activity, and sensory quality at harvest and during storage.

## 2. Materials and Methods

### 2.1. Plant Material and Hydroponic Cultivation

The experiment was conducted in a glasshouse at the University of Pisa, Italy, in the spring of 2022. A weather station was used to track the climate within the glasshouse.

*Salicornia europaea* seeds were purchased from Alsagaden Niederhaslach (France) (https://www.alsagarden.com/, accessed on 10 February 2022) and sown in 240-cell polystyrene trays with stonewool plugs, which were placed in a growth chamber at 25 °C for five days and then transferred to the glasshouse. Four weeks after sowing, the seedlings were transplanted in 50-L hydroponic tanks with stagnant nutrient solution (floating raft system); the water depth in the tanks was 25 cm. Twenty-four plants were grown in each tank, and the crop density was about 96 plants per m^2^. The nutrient solution in each tank was continually aerated, and the concentration of dissolved oxygen was kept above 5.5–6.0 mg/L. Some information about the experiment is reported in Table S1.

In the glasshouse, two different nutrient solutions were compared in a randomized design with three replicates, each consisting of one hydroponic tank with 24 plants: a standard nutrient solution supplemented with 0 g/L of NaCl (C, control) or 12.5 g/L of NaCl (T, treatment). The salt concentration of 12.5 g/L was chosen because it induced the highest growth rate of *Salicornia* plants in a preliminary experiment with NaCl concentrations ranging from 0 to 25 g/L. Both nutrient solutions were prepared by dissolving the appropriate amount of technical-grade inorganic salts in tap water, which contained 0.65 mM Na, to achieve the following nutrient concentration: N (NO_3_) 140.0 mg/L, P 46.5 mg/L, K 352.0 mg/L, Ca 180.5 mg/L, Mg 48.7 mg/L, S (SO_4_) 224.0 mg/L, Fe 2.2 mg/L, B 0.4 mg/L, Cu 0.2 mg/L, Zn 0.7 mg/L, Mn 0.6 mg/L, and Mo 0.1 mg/L. Before use, the pH of all nutrient solutions was adjusted to 5.5 with dilute sulfuric acid. To avoid osmotic shock, the final NaCl concentration was gradually increased during the first week of treatment (the daily increase was 1.78 g/L).

Ninety-four days after sowing, on 17 May 2022, four *Salicornia* plants were collected from each hydroponic tank by cutting the main shoot approximately 1 cm above the collar level. The shoot fresh weight (fw) and dry matter (dm) were then determined, with dm measured after drying the fresh samples in a ventilated oven at 105 °C until a constant weight was achieved [44]. Shoot leaf succulence was calculated as the ratio between shoot water content and dm (expressed as g water/g dm).

### 2.2. Chemical Characterization

#### 2.2.1. Sodium

The concentration of sodium was determined in dry samples [45]. Dried and grinded samples were mineralized with a mixture (5:2) of 65% HNO_3_ and 30% H_2_O_2_ at 240 °C for 1 h. The mineralized samples were used for the determination of the concentration of Na (g/kg dm) by atomic absorption spectroscopy (Varian Model Spectra AA240 FS, Agilent Technologies, Mulgrave, Australia).

#### 2.2.2. Total Polyphenols and Antioxidant Activity

Fresh *Salicornia* samples were characterized for the content of total polyphenols and antioxidant activity. An 80% methanol solution was used to perform a solid/liquid extraction (ratio 1/20 *w*/*v*) from 3 g of a fresh sample, sonicating the mixture in an ultrasonic bath (Elma TI-H-15, Singen, Germany) operating at a frequency of 45 kHz for 30 min at 25 °C. All the extracts were subsequently centrifuged (10 min, 6898 g), filtered on a syringe filter (0.45 μm), and stored at 4 °C until analyses, which were conducted within a few minutes after extraction.

The Folin–Ciocalteu colorimetric method was applied for the total polyphenol spectrophotometric analysis (λ = 765 nm) according to Monacci et al. (2023) [36]; the results were expressed as milligrams of gallic acid equivalents (GAE) per kilogram of dry matter (dm) of sample, according to a 20-point standard curve of GAE in the range 0–2 g/L.

The antioxidant activity of extracts was monitored using the 2,2-diphenyl-1-picrylhydrazyl (DPPH) (λ = 515 nm), the 2,2′-azino-bis(3-ethylbenzothiazoline-6-sulfonic acid) (ABTS) (λ = 734 nm), and the ferric reducing antioxidant power (FRAP) (λ = 593 nm) assays [46]. The results were expressed as µmol Trolox equivalents (TE) per gram of dm, according to different standard curves of TE; the range was 0–200 µmol/L for the DPPH and FRAP assays and 0.1–1.5 mM range for the ABTS assay.

#### 2.2.3. Chlorophylls and Carotenoids

The concentration of chlorophylls and carotenoids was determined spectrophotometrically according to the method described by Lichtenthaler (1987) [47]. Five grams of fresh samples were used to perform a solid/liquid extraction with 20 mL of 100% methanol, sonicating the mixture in an ultrasonic bath (Elma TI-H-15, Singen, Germany) operating at a frequency of 45 kHz for 30 min at 20 °C. The homogenates were centrifuged at 6898× *g* at 4 °C for 10 min in a refrigerated centrifuge (Thermofisher Scientific, Waltham, MA, USA). The absorbance of the supernatant was read at 665.2 nm (A_665.2_), 652.4 nm (A_652.4_), and 470.0 nm (A_470_) in a spectrophotometer UV/VIS (Cary 60, Agilent Technologies, Santa Clara, CA, USA). Chlorophyll-a and chlorophyll-b, total chlorophylls, and carotenoids, expressed as (µg/g dm), were estimated using the following equations:

(1) $$Chlorophyll-a(Ca)=16.72{\;A}_{665.2}-9.16{\;A}_{652.4}$$

(2) $$Chlorophyll-b\left(Cb\right)=34.09{\;A}_{652.4}-15.28{\;A}_{665.2}$$

(3) $$Total\;chlorophyll=1.44{\;A}_{665.2}+24.93{\;A}_{652.4}$$

(4) $$Carotenoids=\frac{1000\;A_{470}-1.63\;Ca-104.96\;Cb}{221}$$

### 2.3. Gaseous Atmosphere inside the Packages and Respiratory Activity

A Dansensor^®^ CheckPoint 3 (Ametek Mocon, Brooklyn Park, MN, USA) composed of a CO_2_ infrared sensor and an O_2_ electrochemical sensor was used to measure the gas composition (concentration of CO_2_ (%) and O_2_ (%)) inside each pack during storage: this information, as previously reported [29,36], permits to assess the respiratory activity. The gas sampling time was 15 s, the O_2_/CO_2_ resolution was 0.1 vol%, and the sensor accuracy was <±0.25 vol%.

### 2.4. Color Determination

The evaluation of the chromatic characteristics of *S. europaea* shoots at harvest and during storage was determined using a Benchtop CLM-196 colorimeter (Eoptis, Trento, Italy). The color values were expressed using the native CIE Lab (L*, a*, b*) coordinates and the calculated cylindrical coordinates (Chroma* and hue*) according to Modesti et al. (2021) [48].

### 2.5. Shelf Life Assessment

*Salicornia* shoots were packed separately in plastic bags by means of an industrial packing machine (Lavezzini 450 GAS, Fiorenzuola d’Arda, Piacenza, Italy). The composition of the internal atmosphere was modified with 100% technical air (78% N_2_, 21% O_2_) and 100% MAP (70% Ar, 23% CO_2_, 7% O_2_). The bags were stored at a temperature-controlled cabinet (4 °C and 20 °C) during the whole observation period (up to 120 h). Thirty packages for each type, each containing 100 g of *Salicornia europaea* grown under 2 salinity levels (C = control (0 g/L of NaCl) and T = treatment (12.5 g/L of NaCl), were set up. Analytical tests were carried out every 24 h on the samples (three packages were opened on each sampling day for each test) to assess the variation in shoot quality.

The samples were weighed daily to assess the weight loss associated with water evaporation during storage; the value was expressed as a percentage reduction compared to the starting value [44].

### 2.6. Sensory Analysis

The sensory profiles were evaluated by a panel of eight trained judges (aged between 23 and 60 years) from the Department of Agriculture, Food, and Environment Sciences at the University of Pisa.

The evaluation of the *Salicornia europaea* shoots at harvest was performed after the definition of a sensory sheet for this product via a preliminary consensus panel and using the sensory parameters reported in Figure S1.

The tasting was also carried out during storage according to the previously developed protocol [2] to assess its evolution over time using only hedonic parameters (visual attractiveness, olfactory pleasantness, taste pleasantness, global acceptability), and on the basis of these, the overall Hedonic Index (HI) was calculated as reported by Bianchi et al. (2022) [29] and used as an index for sensory shelf life of stored products [2]. Each experimental run was terminated when the HI dropped below 6. The research obtained the approval of the ethical committee of the University of Pisa (Comitato Bioetico dell’Università di Pisa, protocol n. 0088081/2022), and informed consent was also obtained from each taster according to the ethical guidelines.

### 2.7. Statistical Analysis

The physical chemical parameters were determined in triplicate, and the significance differences among the means were carried out by one-way ANOVA (CoStat, Version 6.451, CoHort Software, Pacific Grove, CA, USA), and Duncan’s MRT, *p* ≤ 0.05 significance, was used for the separation of the samples.

The results of the sensory analysis were processed by the Big Sensory Soft 2.0 software (version 2018). Sensory data were analyzed by a two-way ANOVA with panelists and samples taken as main factors [29].

The figures were elaborated with the JMP software package version 17 (SAS Institute, Cary, NC, USA).

## 3. Results and Discussion

### 3.1. Effect of Salinity Level

#### 3.1.1. Physical-Chemical Parameters at Harvest

As reported in Table 1, the physical and chemical properties of *S. europaea* plants grown in a hydroponic system changed when the salinity level was 0 g/L or 12.5 g/L.

During plant growth, the salinity level significantly affected the dry matter of shoots at harvesting due to the salt’s retention capacity, with the lowest value reported when the salinity level was 12.5 g/L (Table 1). Therefore, in these conditions, the fresh weight and succulence were the highest in sample T.

Furthermore, *S. europaea* grown with NaCl-enriched nutrient solution showed the lowest polyphenol content and antioxidant activity but the highest level of chlorophylls and carotenoids.

Further, as the salinity level was applied, the brightness of the color increased, together with the color saturation in green tones and the overall color intensity.

#### 3.1.2. Sensory Evaluation at Harvest

**Figure 1:**
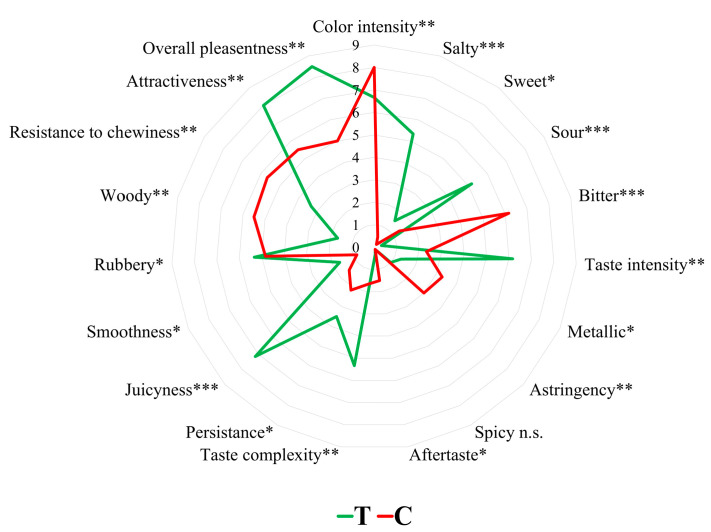
Sensory parameters evaluated at harvest in *Salicornia europaea* shoots with different salinity levels (0 g/L NaCl (C) and 12.5 g/L NaCl (T)). Significance level: *** = *p* < 0.001; ** = *p* < 0.01; * = *p* < 0.05; n.s. = not significant (*p* ≥ 0.05).

At harvest time, the sensory profile of *S. europaea* shoots exhibited significant differences across a wide range of organoleptic parameters due to the application of NaCl-enriched nutrient solution (Figure 1), with taste profile and tactile sensations significantly improved. This improvement included higher taste intensity and complexity, along with heightened saltiness, sourness, and juiciness, while bitterness and woody sensations showed a significant reduction. As a result, both overall pleasantness and attractiveness were significantly enhanced in salt-treated plants (T). This positive effect on sensory perception confirmed the reliability of the salinity regimen implemented during hydroponic cultivation in enhancing the taste experience of *S. europaea*.

### 3.2. Shelf Life in Different Packaging Conditions

As *S. europaea* is generally easily perishable, selecting efficient storage methods is critical to minimizing post-harvest losses as well as preserving as long as possible during storage the initial nutritional values, color appearance, and sensory profile for consumer acceptance. The following sections will explore the factors that affected the shelf life of *S. europaea* as a function of cultivation and storage conditions, to select proper conditions to keep it fresh for as long as possible and maintain its good appearance from farm to customer.

According to Custódio et al. (2021) [49], the proximate composition of some *Salicornia* has a high moisture content, generally above 80%. As a result, the moisture level has an impact on the shelf life and consumption quality of fresh vegetables, as well as food safety from foodborne pathogens [50]. In this experiment, we explored the hydroponically grown conditions (salinity), packaging conditions, and storage temperatures. After that, we investigated the factors that affected the shelf life of *S. europaea*, including the percentage of dry matter, biochemical compositions, color parameters, antioxidant capacities, weight loss, respiratory activities, and sensory evolution.

#### 3.2.1. Dry Matter Content

Generally speaking, the dry matter in plant material consists of all constituents, excluding water. The dry matter content of edible plants was a good predictor of their quality, which is generally associated with their content in carbohydrates, fats, proteins, vitamins, minerals, and antioxidants [51].

As reported above, the dry matter percentage of *S. europaea* was significantly affected by the salinity level of the nutrient solution, the storage temperature, and the packaging with MAP. As expected, the results reported in Table 2 showed that in all the experimental runs, the dry matter content increased significantly during storage, with some significant differences among samples. More in depth, after 96 h of storage, at the same salinity level used for plant growing, dry matter was not affected by storage conditions (*p* > 0.05), with the lowest values still reported in group T. This result aligned with the biochemical compositions, chlorophyll content, and antioxidant activity, according to Amoruso et al. (2022) [52]. They found that a salinity of 9 mM NaCl had a more significant impact on the growth, quality, and shelf life of fresh-cut sea fennel plants (*Crithmum maritimum* L.) in a hydroponic floating system than a salinity of 150 mM NaCl, as the aerial part increased fresh weight due to salt stress. On the other hand, group T showed the lowest values of dry matter regardless of both storage temperature and gas composition, while the highest one was observed for control samples stored at 20 °C.

With all other experimental conditions remaining constant, MAP does not seem to reduce the concentration process when compared with air.

#### 3.2.2. Weight Loss and Respiratory Activity

**Figure 2:**
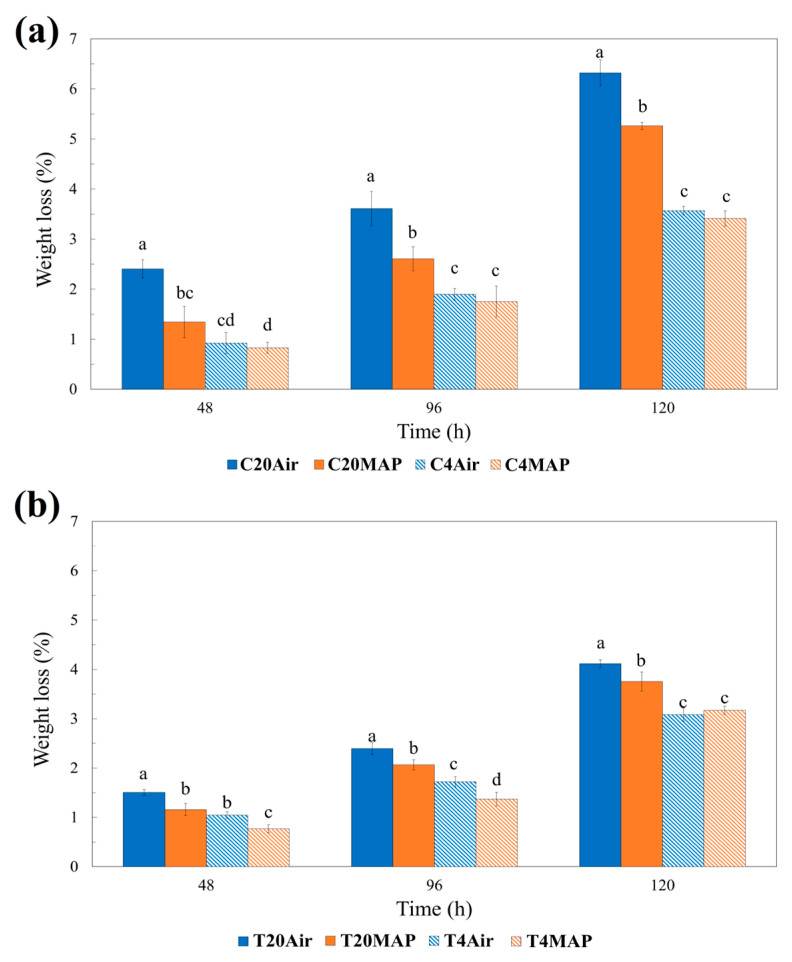
Weight loss during storage time at different temperatures (4 °C and 20 °C) and packaging atmospheres (Air and MAP) of *Salicornia europaea* shoots with different salinity levels: (**a**) C (0 g/L NaCl); (**b**) T (12.5 g/L NaCl).Values are the average (bars indicate ± SD) (n = 3). Different letters in each group indicate statistical differences (Duncan’s MRT, *p* ≤ 0.05).

Figure 2a,b show the trend of weight loss during time of *S. europaea* as a function of growing and storage conditions. As a result, the highest weight loss was observed at room temperature for C shoots stored in air, closely followed by the same plants stored in MAP, while the salt nutrition and refrigeration counteracted this phenomenon in all the operating conditions adopted.

This outcome underscores the advantages of using MAP for storage, as it effectively slowed down physiological processes that contribute to weight loss, such as respiration and transpiration, especially for samples stored at room temperature.

This result aligned with the findings of Lu et al. (2010) [19], who found that storing *S. bigelovii* at a temperature lower than 8 °C could sustain the weight loss percentage. This was due to the high physiological metabolism and rapid senescence caused by high temperature storage (25 °C), which led to significant weight loss in *S. bigelovii* and caused the sample tissue to wilt.

The enhanced moisture retention in MAP likely plays a critical role in preserving the structural integrity of *S. europaea*, thus minimizing weight loss. Moreover, the potential economic benefits of reduced weight loss and improved quality could lead to increased consumer satisfaction and demand.

**Figure 3:**
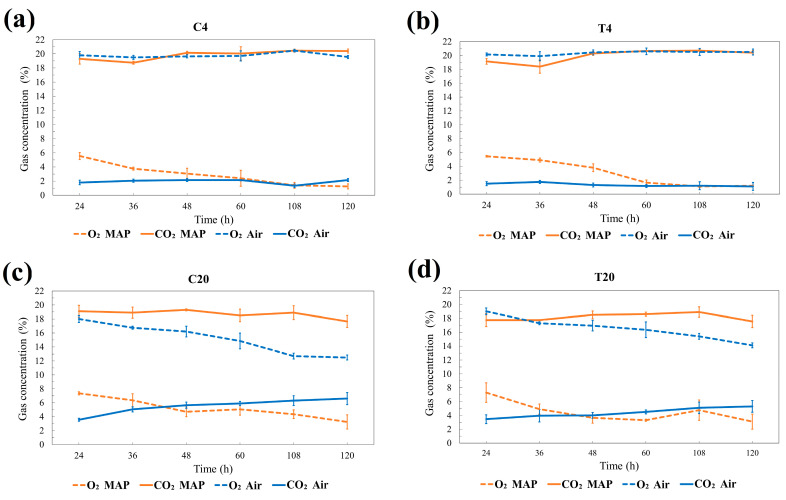
Gas concentration (% O_2_ and % CO_2_) inside package during storage at different packaging conditions (Air and MAP) of *Salicornia europaea* shoots with different salinity levels: (**a**) 0 g/L stored at 4 °C (C4); (**b**) 12.5 g/L stored at 4 °C (T4); (**c**) 0 g/L stored at 20 °C (C20); (**d**) 12.5 g/L stored at 20 °C (T20). Values are the average (bars indicate ± SD) (n = 3).

Shelf life often correlates with respiratory rate, as it can be used as an index for metabolic activity. *Salicornia* is highly perishable, with a short shelf life under ambient conditions. Therefore, reduced respiration has a key role in retarding *Salicornia* spoilage after harvest [53]. Figure 3a–d represent the results of the respiratory rate of *S. europaea*, which was grown at different salinity levels and stored at different temperatures and packaging conditions.

As expected, when *S. europaea* was stored in ambient air at 20 °C, we observed a significant decline in O_2_ levels, which decreased from an initial concentration of 20% to 16% after 120 h, alongside an increase in CO_2_ concentrations, rising from 2% to 7%. This data indicated that the plants consumed the available O_2_ for respiration, resulting in elevated CO_2_ and vapor levels within the plastic bag. This process not only produces energy but also can allow the production of off-odors, resulting from the accumulation of fermentation by-products and the subsequent deterioration of the fresh produce [35]. In contrast, when *S. europaea* was stored at 4 °C in air (as shown in Figure 3a,b), the gas concentration trends revealed that O_2_ levels remain relatively stable, fluctuating minimally from the initial measurement. This stability suggested that the plants underwent a significantly reduced rate of CO_2_ production during respiration, with the concentration of CO_2_ remaining stable. The implications of this reduced respiratory activity were substantial; lower storage temperatures effectively diminished the plant’s metabolic processes, slowing down the accumulation of CO_2_ and delaying the spoilage of fresh products [43].

On the other hand, the application of MAP for packaging could be an effective solution to reduce respiratory activity during storage even without refrigeration, with the best results shown by *S. europaea* grown in salinity conditions (T).

As the modified atmosphere reduced oxygen levels and slowed down the metabolic rate, thereby extending the shelf life of the product even without refrigeration, growers and distributors should consider adopting MAP to reduce the energetic costs while preserving the product’s marketability and shelf life. Further studies should be useful to verify the effectiveness of the use of MAP combined with intermediate temperatures (i.e., 8/10 °C) for storage.

#### 3.2.3. Biochemical Compositions

Table 3 and Table S1 summarize the biochemical compositions of *S. europaea*, grown in C and T at different temperatures and packaging conditions, after 120 h of harvest. The results indicated total chlorophylls, carotenoids, and total polyphenols.

Generally, photosynthetic organisms in chloroplasts contain chlorophyll, a natural green pigment. Some parts of vegetables and fruits also contain chlorophyll, which contributes to the increase of antioxidants in the human bloodstream [54]. Additionally, the color parameters of *Salicornia*, which consumers use to determine the freshness of this plant, correlate with chlorophyll. The preservation of chlorophyll not only maintained the plant’s visual quality but also enhanced its nutritional profile, making this a critical finding for post-harvest management [55,56].

Carotenoids and polyphenols are compounds derived from plants that have been proven to show powerful antioxidant and anti-inflammatory properties [57]. Carotenoids, such as chlorophyll, are sensitive to environmental conditions, and their retention during storage is essential for maintaining the health benefits of *S. europaea*.

As reported in Table 3, storing *S. europaea* at 4 °C could maintain those compounds better than at 20 °C, with results similar to those of Lu et al. (2010) [19]. Further, MAP allowed to slow down the degradation of chlorophyll, carotenoids, and total phenols even at room temperature, with the major effect observed for T samples.

#### 3.2.4. Antioxidant Capacity

Fruits and vegetables typically lose some of their antioxidant capacity when storage temperature and/or time increase [58]. Table 4 presents the changes in the primary antioxidant capacity of *S. europaea* after harvesting for 120 h. As a result, MAP helped to preserve the antioxidant capacity of samples during storage, with best results at 4 °C, while the salinity levels of the nutrient solution had no significant effect.

According to Poljsak et al. (2021) [59], catalysts and oxidase enzymes such as polyphenol oxidase (PPO) accelerate oxidation, especially at higher temperatures, leading to a decrease in antioxidant capabilities. In this study, storage at 4 °C was the most effective for maintaining the quality of *S. europaea*.

This finding highlighted that temperature played a more critical role than salinity in the post-harvest preservation of antioxidants.

The unexpected lack of influence from the salinity levels on antioxidant activity deserves further exploration. While previous studies suggest that salt stress can induce antioxidant production by enhancing the activity of reactive oxygen species (ROS) scavengers, this effect may be less pronounced post-harvest or under the specific conditions of this study. It would be valuable to investigate whether different harvest times or salinity levels could produce varying outcomes or if the antioxidant degradation observed was due to a rapid post-harvest enzyme-driven process that overshadowed any pre-existing effects from salt stress.

#### 3.2.5. Color Parameters

The color parameters L*, a*, b*, chroma*, and hue* are widely used to evaluate food color, as they significantly influence consumer acceptance [60]. These parameters are essential indicators of visual quality and consumer perception of freshness in food products, making their preservation critical in post-harvest handling. Table 5 and Table S2 provide an overview of these color parameters during storage of *S. europaea* shoots at different temperatures and packaging conditions.

The parameter L* indicates brightness, ranging from 0 (black) to 100 (white). The a* and b* values represent chromaticity: negative a* values denote green, while positive values represent red; negative b* values indicate blue, while positive values reflect yellow. C* measures chroma, or color intensity, while h* indicates hue angle, which specifies the exact color [60].

In this study, *S. europaea* stored under MAP maintained its color parameters significantly better than those stored in Air (*p* ≤ 0.05). MAP reduced color and pigment decay by limiting their oxidation and browning [61,62].Oxygen, in particular, promotes the enzymatic browning of fresh produce through the activity of polyphenol oxidase (PPO), which catalyzes the oxidation of phenolic compounds, leading to discoloration. By reducing oxygen levels, MAP minimizes oxidative stress, thereby preserving the chlorophyll and carotenoid pigments that give *S. europaea* its vibrant color [63].

Additionally, the temperature of 4 °C, compared to 20 °C, better preserved the brightness and saturation of *S. europaea* while reducing oxidation and browning. Lower temperatures slowed down enzymatic reactions, including those that caused pigment degradation and browning. Higher temperatures, in contrast, accelerated the breakdown of chlorophyll and other pigments, resulting in loss of color and visual appeal.

Furthermore, the positive effect of MAP in preserving color attributes was most evident for T shoots stored at room temperature.

Further research into the interactions between color retention, oxidative stress, and packaging technologies could also offer valuable insights for optimizing post-harvest storage methods.

### 3.3. Sensory Evaluation during Storage

In Figure S2a,b, the temporal trends of the Hedonic Index (HI) during the storage period are shown as a function of both the salinity level used and the following applied storage conditions. Although the trends were calculated based on a limited number of experimental points, it seems possible to identify a consistent trend regardless of the salinity level used during cultivation. In greater detail, under the same storage atmosphere, the sensory acceptability limit was reached more quickly when plants were stored at 20 °C, while a higher degradation rate was observed in the presence of air regardless of the storage temperature applied.

**Figure 4:**
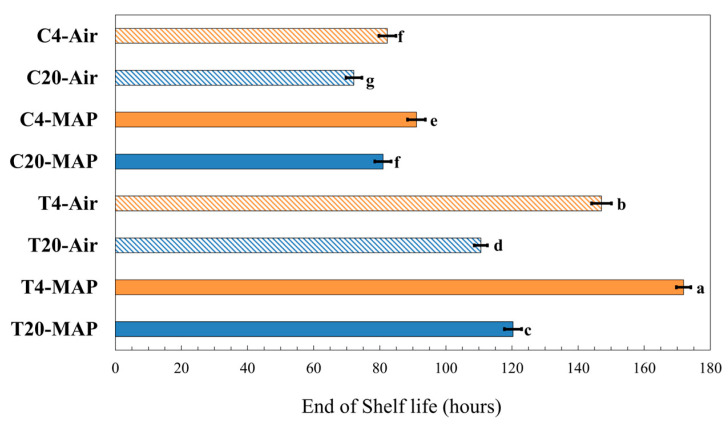
Time (hours) of shelf life end, which the Hedonic Index falls below the acceptability limit (HI ≤ 6) in *Salicornia europaea* grown in the hydroponic system with different salinity levels (0 g/L (C) and 12.5 g/L (T)) stored at two temperatures (4 °C and 20 °C) and with two packaging atmospheres (Air and MAP). Values are the average (bars indicate ± SD) (n = 3). Different letters indicate significant differences in value (Duncan’s MRT, *p* ≤ 0.05).

Figure 4 illustrated the sensory shelf life, during which the HI fell below the acceptability limit (HI ≤ 6) across different experimental runs [2]; the time (hours) of the end of shelf life was calculated based on the equations obtained for the line regression of the HI as reported in Figure S2a,b. Irrespective of the storage conditions, the sensory shelf life increased in each experimental run when a salinity level was applied during hydroponic cultivation. Furthermore, the temperature had the greatest effect on the *Salicornia* shelf life, with storage at 4 °C compared to 20 °C showing the best results in terms of preservation.

As far as the packaging atmosphere was concerned, different results were obtained. When using glasswort shoots grown in saline solution (T) stored in MAP, the shelf life increased considerably regardless of the stored temperature, while the same effect was not obtained for C, where there are no statistical differences between Air and MAP (Figure 4).

All in all, the best results in terms of sensory shelf life were obtained when *Salicornia europaea* was grown in saline-nutrient solution and stored at 4 °C in a modified atmosphere packaging.

The time of 170 h (about 7 days) was obtained in T4-MAP and confirmed that it is the optimal storage condition to be used because the shelf life is maximized. This is in line with what has been reported in the literature [19,28]: at room temperature in the air, the duration never exceeded 6 days.

## 4. Conclusions

Halophytes, particularly *S. europaea*, could help address challenges posed by aridity and climate change. Integrating aquaponics for the simultaneous cultivation of agricultural species represents an effective strategy to tackle environmental, social, and economic issues. However, since *S. europaea* is highly perishable, identifying the best conditions for its cultivation and storage is crucial to minimize economic losses from waste production.

To this extent, in the experimental conditions adopted, salinity levels significantly influenced both *S. europaea* quality at harvesting and post-harvest shelf life. At harvesting, cultivating plants in a saline nutrient solution at 12.5 g/L NaCl resulted in enhanced biochemical, antioxidant, and organoleptic profiles. During storage, plants grown in salinity showed an increase in water retention capability and a consequent reduction in weight loss over time, together with improved sensory attributes such as taste and tactile sensations as well as color intensity.

Besides, storage temperature and packaging method also significantly influenced shoot respiration rates and sensory qualities. As expected, storage in air at higher temperatures (20 °C) accelerated weight loss and diminished color stability, particularly in no-salinity samples, while MAP extended the shelf life of all the samples regardless of the storage temperature adopted. Optimal storage conditions were observed when MAP was combined with refrigeration, which allowed for effectively preserving plant sensory acceptability over a period of about seven days.

Lastly, optimizing salinity during hydroponic cultivation, alongside appropriate storage conditions, can significantly improve the shelf life and sensory quality of *S. europaea*, offering valuable insights for its commercial viability.

Overall, these results hold some interesting implications for both growers and distributors. By storing *S. europaea* under MAP conditions, producers can ensure better preservation of key antioxidants, enhancing the plant’s nutritional value and shelf life even at temperatures higher than 4 °C. This could potentially improve consumer satisfaction and increase the commercial appeal of *S. europaea* by retaining its biochemical quality during storage. Furthermore, these optimized storage practices could lead to reduced post-harvest losses while reducing energetic costs related to refrigeration at 4 °C, thus improving the economic viability of *S. europaea* production.

In conclusion, although this study focused on a 120-h observation period, the evaluation of sensory shelf life suggested that under optimal storage conditions, the hedonic index of stored plants can remain above the acceptability threshold for up to 170 h.

Future research could further explore the long-term effects on the nutritional value and sensory quality of *S. europaea* under various combinations of modified atmosphere packaging and different storage temperatures, ranging from 4 °C and 20 °C.

## Figures and Tables

**Table 1:** Effect of salinity level (C = 0 g/L NaCl and T = 12.5 g/L NaCl) on the physical-chemical parameters (reported on dry matter basis) after harvest of *Salicornia europaea* shoots.

Parameters	Units	C	T
Shoot fresh weight	g/plant	103.33 ± 18.45 ^b^	271.80 ± 9.13 ^a^
Dry matter (dm)	%	11.93 ± 0.21 ^a^	8.23 ± 0.18 ^b^
Succulence	g water/g dm	7.38 ± 0.17 ^b^	11.15 ± 0.27 ^a^
Sodium	g/kg dm	12.20 ± 0.20 ^b^	160.41 ± 10.51 ^a^
Total polyphenols	mg GAE/g dm	15.59 ± 0.50 ^a^	14.24 ± 0.21 ^b^
Chlorophyll-a	µg/g dm	889.42 ± 4.30 ^b^	1151.05 ± 6.13 ^a^
Chlorophyll-b	µg/g dm	573.23 ± 8.42 ^b^	722.60 ± 7.84 ^a^
Total chlorophylls	µg/g dm	1462.66 ± 6.36 ^b^	1873.66 ± 6.98 ^a^
Carotenoids	µg/g dm	539.43 ± 10.55 ^b^	702.95 ± 10.63 ^a^
ABTS	µmol TE/g dm	42.20 ± 0.50 ^a^	38.25 ± 0.38 ^b^
DPPH	µmol TE/g dm	29.54 ± 0.35 ^a^	26.78 ± 0.36 ^b^
FRAP	µmol TE/g dm	46.43 ± 0.55 ^a^	42.08 ± 0.49 ^b^
L*		28.36 ± 0.05 ^b^	33.43 ± 0.06 ^a^
a*		−10.32 ± 0.04 ^b^	−12.95 ± 0.06 ^a^
b*		16.51 ± 0.07 ^b^	19.91 ± 0.03 ^a^
C*		19.47 ± 0.08 ^b^	23.75 ± 0.01 ^a^
h*		−58.00 ± 0.02 ^b^	−56.96 ± 0.15 ^a^

Values are presented as the average ± standard deviation (SD) (n = 3). Different letters in the row indicate statistical differences (Duncan’s MRT, *p* ≤ 0.05).

**Table 2:** Effect of temperature (4 °C and 20 °C) and packaging atmosphere (Air and MAP) during storage on the dry matter (%) of *Salicornia europaea* shoots with different salinity levels (C and T).

Salinity Level (g/L NaCl)	Temperature (°C)	Atmosphere	Dry Matter (%)		
			Time (h)		
			48	96	120
0 (C)	4	Air	12.92 ± 0.20 ^A,c^	14.31 ± 0.13 ^A,b^	15.50 ± 0.02 ^B,a^
		MAP	12.91 ± 0.06 ^A,c^	14.20 ± 0.05 ^A,b^	15.48 ± 0.06 ^B,a^
	20	Air	13.08 ± 0.21 ^A,c^	14.13 ± 0.14 ^A,b^	15.79 ± 0.07 ^A,a^
		MAP	12.97 ± 0.10 ^A,c^	14.15 ± 0.11 ^A,b^	15.70 ± 0.08 ^A,a^
12.5 (T)	4	Air	9.25 ± 0.15 ^B,c^	10.54 ± 0.14 ^B,b^	11.85 ± 0.18 ^C,a^
		MAP	9.23 ± 0.07 ^B,c^	10.51 ± 0.05 ^B,b^	11.80 ± 0.07 ^C,a^
	20	Air	9.42 ± 0.16 ^B,c^	10.48 ± 0.04 ^B,b^	11.98 ± 0.16 ^C,a^
		MAP	9.36 ± 0.11 ^B,c^	10.45 ± 0.05 ^B,b^	11.90 ± 0.12 ^C,a^

Values are presented as average ± standard deviation (SD) (n = 3). ^A–C^ Different letter superscripts are significantly different within columns (Duncan’s MRT, *p* ≤ 0.05). ^a–c^ Different letter superscripts are significantly different within rows (Duncan’s MRT, *p* ≤ 0.05).

**Table 3:** Effect of temperature (4 °C and 20 °C) and packaging atmosphere (Air and MAP) during storage on total chlorophylls (µg/g dm), carotenoids (µg/g dm), and total polyphenols (mg GAE/g dm) content of *Salicornia europaea* shoots with different salinity levels (C and T).

Salinity Level (g/L NaCl)	Temperature (°C)	Atmosphere	Total Chlorophylls (µg/g dm)			Carotenoids (µg/g dm)			Total Polyphenols (mg GAE/g dm)		
			Time (h)			Time (h)			Time (h)		
			48	96	120	48	96	120	48	96	120
0 (C)	4	Air	1046.25 ± 1.58 ^E,a^	818.86 ± 4.85 ^D,b^	664.12 ± 7.81 ^F,c^	437.80 ± 14.85 ^E,a^	289.02 ± 9.30 ^E,b^	217.48 ± 2.03 ^E,c^	9.24 ± 0.12 ^C,a^	7.28 ± 0.07 ^D,b^	6.15 ± 0.14 ^D,c^
		MAP	1125.25 ± 9.48 ^D,a^	952.92 ± 9.38 ^C,b^	739.27 ± 6.91 ^D,c^	487.22 ± 7.16 ^C,a^	326.34 ± 1.75 ^D,b^	235.98 ± 12.86 ^D,c^	9.47 ± 0.30 ^BC,a^	7.34 ± 0.14 ^D,b^	6.41 ± 0.06 ^C,c^
	20	Air	1042.08 ± 14.63 ^EF,a^	800.63 ± 9.42 ^E,b^	657.10 ± 4.16 ^F,c^	443.50 ± 11.25 ^E,a^	272.79 ± 5.48 ^F,b^	196.57 ± 1.70 ^F,c^	8.91 ± 0.17 ^D,a^	6.68 ± 0.13 ^E,b^	5.85 ± 0.12 ^E,c^
		MAP	1029.70 ± 8.28 ^F,a^	805.20 ± 12.05 ^DE,b^	715.48 ± 8.65 ^E,c^	460.09 ± 2.57 ^D,a^	287.55 ± 3.49 ^E,b^	205.02 ± 8.84 ^E,c^	8.89 ± 0.27 ^D,a^	7.39 ± 0.09 ^D,b^	5.92 ± 0.05 ^E,c^
12.5 (T)	4	Air	1260.50 ± 9.92 ^B,a^	1020.05 ± 3.76 ^B,b^	824.88 ± 5.11 ^B,c^	578.35 ± 7.69 ^A,a^	388.85 ± 6.24 ^B,b^	269.51 ± 2.47 ^B,c^	10.73 ± 0.22 ^A,a^	8.35 ± 0.04 ^B,b^	6.86 ± 0.11 ^AB,c^
		MAP	1311.04 ± 26.70 ^A,a^	1100.78 ± 28.86 ^A,b^	881.77 ± 12.83 ^A,c^	572.37 ± 2.01 ^A,a^	440.99 ± 4.92 ^A,b^	306.51 ± 1.45 ^A,c^	10.85 ± 0.37 ^A,a^	9.20 ± 0.24 ^A,b^	6.97 ± 0.075 ^A,c^
	20	Air	1227.75 ± 14.42 ^C,a^	968.18 ± 16.37 ^C,b^	767.34 ± 11.90 ^C,c^	534.57 ± 10.70 ^B,a^	343.66 ± 6.15 ^C,b^	191.31 ± 11.52 ^F,c^	9.52 ± 0.12 ^C,a^	8.02 ± 0.11 ^C,b^	6.62 ± 0.07 ^B,c^
		MAP	1246.51 ± 10.46 ^B,a^	1031.60 ± 11.19 ^B,b^	762.34 ± 9.41 ^C,c^	518.34 ± 9.53 ^B,a^	376.61 ± 7.46 ^B,b^	259.10 ± 1.30 ^C,c^	9.88 ± 0.12 ^B,a^	8.56 ± 0.15 ^B,b^	6.91 ± 0.15 ^A,c^

Values are presented as average ± standard deviation (SD) (n = 3). ^A–F^ Different superscript uppercase letters indicate significant differences within columns (Duncan’s MRT, *p* ≤ 0.05). ^a–c^ Different superscript lowercase letters indicate significant differences within rows (Duncan’s MRT, *p* ≤ 0.05).

**Table 4:** Effect of temperature (4 °C and 20 °C) and packaging atmosphere (Air and MAP) during storage on the antioxidant capacity (ABTS, DPPH, and FRAP) expressed as µmol Trolox equivalents (TE) per gram of dry matter (dm) of *Salicornia europaea* shoots with different salinity levels (C and T).

Salinity Level (g/L NaCl)	Temperature (°C)	Atmosphere	ABTS (µmol TE/g dm)			DPPH (µmol TE/g dm)			FRAP (µmol TE/g dm)		
			Time (h)			Time (h)			Time (h)		
			48	96	120	48	96	120	48	96	120
0 (C)	4	Air	25.18 ± 0.85 ^E,a^	19.98 ± 0.23 ^E,b^	16.18 ± 0.41 ^E,c^	17.62 ± 0.40 ^E,a^	13.99 ± 0.22 ^E,b^	11.33 ± 0.50 ^CD,c^	27.69 ± 0.64 ^F,a^	21.65 ± 0.69 ^E,b^	17.80 ± 0.39 ^E,c^
		MAP	26.87 ± 0.82 ^D,a^	21.68 ± 0.29 ^D,b^	17.16 ± 0.28 ^D,c^	18.81 ± 0.58 ^D,a^	15.17 ± 0.20 ^D,b^	12.01 ± 0.20 ^C,c^	29.55 ± 0.51 ^E,a^	23.84 ± 0.32 ^D,b^	18.87 ± 0.31 ^D,c^
	20	Air	23.87 ± 0.50 ^F,a^	19.68 ± 0.63 ^E,b^	15.28 ± 0.12 ^F,c^	16.71 ± 0.35 ^F,a^	13.49 ± 0.25 ^F,b^	10.94 ± 0.19 ^D,c^	25.06 ± 0.55 ^G,a^	20.23 ± 0.86 ^F,b^	16.41 ± 0.30 ^F,c^
		MAP	24.07 ± 0.76 ^EF,a^	19.27 ± 0.78 ^E,b^	15.63 ± 0.28 ^EF,c^	16.85 ± 0.53 ^F,a^	13.78 ± 0.44 ^EF,b^	10.69 ± 0.18^D,c^	26.48 ± 0.84 ^G,a^	21.98 ± 0.23 ^E,b^	16.80 ± 0.33 ^F,c^
12.5 (T)	4	Air	35.04 ± 0.85 ^A,a^	26.09 ± 0.75 ^B,b^	20.79 ± 0.22 ^AB,c^	22.91 ± 0.30 ^B,a^	18.26 ± 0.53 ^B,b^	14.55 ± 0.21 ^A,c^	37.00 ± 0.57 ^B,a^	28.70 ± 0.83 ^B,b^	22.87 ± 0.23 ^AB,c^
		MAP	35.24 ± 0.51 ^A,a^	28.70 ± 0.33 ^A,b^	21.08 ± 0.65 ^A,c^	24.67 ± 0.36 ^A,a^	20.09 ± 0.93 ^A,b^	14.75 ± 0.66 ^A,c^	38.77 ± 0.24 ^A,a^	30.14 ± 0.46 ^A,b^	23.19 ± 0.24 ^A,c^
	20	Air	31.65 ± 0.39 ^C,a^	24.00 ± 0.47 ^C,b^	19.23 ± 0.91 ^C,c^	22.16 ± 0.18 ^C,a^	15.60 ± 0.78 ^C,b^	13.46 ± 0.64^B,c^	34.82 ± 0.43 ^D,a^	26.40 ± 0.38 ^C,b^	20.20 ± 0.21 ^C,c^
		MAP	32.62 ± 0.30 ^B,a^	25.75 ± 0.80 ^B,b^	20.59 ± 0.28 ^B,c^	22.83 ± 0.21 ^B,a^	18.02 ± 0.56 ^B,b^	13.38 ± 0.76 ^B,c^	35.88 ± 0.33 ^C,a^	28.32 ± 0.88 ^B,b^	22.65 ± 0.29 ^B,c^

Values are presented as average ± standard deviation (SD) (n = 3). ^A–G^ Different superscript uppercase letters indicate significant different values within columns (Duncan’s MRT, *p* ≤ 0.05). ^a–c^ Different superscript lowercase letters indicate significant differences within rows (Duncan’s MRT, *p* ≤ 0.05).

**Table 5:** Effect of temperature (4 °C and 20 °C) and packaging atmosphere (Air and MAP) during storage on color parameters (L*, a*, and b*) of *Salicornia europaea* shoots with different salinity levels (C and T).

Salinity Level (g/L)	Temperature (°C)	Atmosphere	L*			a*			b*		
			Time (h)			Time (h)			Time (h)		
			48	96	120	48	96	120	48	96	120
0 (C)	4	Air	24.06 ± 1.04 ^C,c^	31.25 ± 0.05 ^AB,a^	27.33 ± 1.31 ^AB,b^	−12.26 ± 1.06 ^A,a^	−12.58 ± 0.31 ^A,a^	−13.03 ± 0.46 ^AB,a^	15.86 ± 1.53 ^C,b^	18.23 ± 0.18 ^C,a^	19.06 ± 1.02 ^A,a^
		MAP	27.62 ± 1.12 ^B,b^	33.54 ± 0.11 ^A,a^	23.91 ± 0.04 ^AB,c^	−12.81 ± 0.75 ^A,b^	−14.28 ± 0.23 ^A,a^	−10.94 ± 0.79 ^ABC,c^	17.81 ± 0.55 ^ABC,b^	22.00 ± 0.23 ^AB,a^	13.94 ± 1.08 ^BC,c^
	20	Air	28.56 ± 0.43 ^B,a^	27.95 ± 0.03 ^CD,b^	23.62 ± 1.13 ^B,c^	−12.64 ± 0.33 ^A,a^	−12.25 ± 0.09 ^A,a^	−10.08 ± 1.02 ^ABC,b^	16.43 ± 1.66 ^BC,b^	18.93 ± 0.05 ^BC,a^	17.29 ± 1.17 ^AB,ab^
		MAP	31.10 ± 0.16 ^A,a^	29.88 ± 0.06 ^BCD,b^	19.33 ± 1.80 ^C,c^	−14.07 ± 0.13 ^A,a^	−13.01 ± 0.12 ^A,b^	−7.67 ± 1.51 ^C,c^	20.23 ± 0.42 ^AB,b^	22.40 ± 0.16 ^A,a^	11.29 ± 1.16 ^C,c^
12.5 (T)	4	Air	28.21 ± 0.18 ^B,b^	30.78 ± 1.27 ^ABC,a^	28.40 ± 0.26 ^A,b^	−13.87 ± 0.23 ^A,a^	−13.54 ± 0.39 ^A,a^	−13.78 ± 0.57 ^A,a^	18.22 ± 1.42 ^ABC,a^	18.95 ± 1.58 ^BC,a^	20.57 ± 1.27 ^A,a^
		MAP	30.37 ± 1.12 ^AB,a^	23.47 ± 1.56 ^E,b^	17.82 ± 1.30 ^C,c^	−13.97 ± 0.09 ^A,a^	−11.79 ± 0.32 ^A,b^	−9.97 ± 0.88 ^BC,c^	21.83 ± 0.28 ^A,a^	18.03 ± 1.23 ^C,b^	13.67 ± 1.12 ^BC,c^
	20	Air	30.16 ± 1.07 ^B,a^	27.43 ± 1.67 ^D,b^	17.98 ± 1.33 ^C,c^	−14.24 ± 1.31 ^A,a^	−12.39 ± 1.94 ^A,a^	−9.57 ± 1.87 ^BC,b^	19.90 ± 1.27 ^ABC,a^	20.35 ± 0.94 ^ABC,a^	14.56 ± 1.60 ^BC,b^
		MAP	30.49 ± 0.57 ^AB,b^	31.96 ± 0.14 ^AB,a^	26.37 ± 1.94 ^AB,c^	−14.18 ± 0.68 ^A,a^	−13.44 ± 0.36 ^A,a^	−11.46 ± 0.48 ^AB,b^	18.73 ± 1.35 ^ABC,a^	20.13 ± 1.41 ^ABC,a^	19.26 ± 1.08 ^A,a^

Values are presented as average ± standard deviation (SD) (n = 3). ^A–E^ Different superscript uppercase letters indicate significant different values within columns (Duncan’s MRT, *p* ≤ 0.05). ^a–c^ Different superscript lowercase letters indicate significant differences within rows (Duncan’s MRT, *p* ≤ 0.05).

## Data Availability

The original contributions presented in the study are included in the article and Supplementary Materials, further inquiries can be directed to the corresponding author.

## Supplementary Materials

The following supporting information can be downloaded at: https://www.mdpi.com/article/10.3390/foods13203260/s1. Table S1: Basic information on the experiment with *Salicornia europaea* plants grown in a floating raft system in a glasshouse in 2022; Table S2: Effect of temperature (4 °C and 20 °C) and packaging atmosphere (Air and MAP) during storage on chlorophyll-a and -b content (µg/g dm) of *Salicornia europaea* shoots with different salinity levels (C and T); Table S3: Effect of temperature (4 °C and 20 °C) and packaging atmosphere (Air and MAP) during storage on color parameters (Chroma* and Hue*) of *Salicornia europaea* shoots with different salinity levels (C and T); Figure S1: Sensory parameters selected and evaluated at harvest in *Salicornia europaea* shoots grown in hydroponic systems with different salinity levels (C and T); Figure S2: Line regression of the trend of hedonic index (HI) during storage at different temperatures (4 °C and 20 °C) and packaging atmospheres (Air and MAP) of *Salicornia europaea* shoots grown in hydroponic systems with different salinity levels: (a) C (0 g/L NaCl); (b) T (12.5 g/L NaCl). Values are the average (bars indicate ± SD) (n = 3).
