# Supplementary material for: Effects of Temperature and Packaging Atmosphere on Shelf Life, Biochemical, and Sensory Attributes of Glasswort (Salicornia europaea L.) Grown Hydroponically at Different Salinity Levels
Source: Foods. 2024 Oct 13;13(20):3260. doi: 10.3390/foods13203260 (PMC11507112; doi:10.3390/foods13203260)
Supplement: Supplementary file 1 [file foods-13-03260-s001.zip › foods-3225106-supplementary.pdf]

Table S1. Basic information on the experiment with *Salicornia europaea* plants grown in a floating raft system in a glasshouse in 2022.

Sowing date	11 February
Transplant date	11 March
Start of treatment	24 March
Harvest date	17 May
Days of treatment	54
Mean air temperature (°C)	20.5*
Mean daily solar radiation (MJ m^2/day)	8.49*
Cumulative solar radiation (MJ m^2)	577.59*

* The values were computed for the period from transplanting to harvest.

Table S2. Effect of temperature (4 °C and 20 °C) and packaging atmosphere (Air and MAP) during storage on chlorophyll-a and -b (μg/g dm) of *Salicornia europaea* shoots with different salinity levels (C and T).

Salinity level (g/L NaCl)	Temperature (°C)	Atmosphere	Chlorophyll-a (μg/g dm)			Chlorophyll-b (μg/g dm)		
			Time (h)			Time (h)		
			48	96	120	48	96	120
0 (C)	4	Air	627.29 ± 9.42 ^{E,a}	445.67 ± 3.59 ^{D,b}	395.37 ± 12.75 ^{D,c}	432.33 ± 1.72 ^{D,a}	373.19 ± 6.10 ^{C,b}	320.11 ± 4.56 ^{D,c}
		MAP	666.97 ± 7.97 ^{D,a}	535.85 ± 14.32 ^{C,b}	402.34 ± 6.21 ^{CD,c}	458.28 ± 10.99 ^{C,a}	417.07 ± 4.44 ^{B,b}	336.92 ± 7.62 ^{C,c}
	20	Air	613.92 ± 1.43 ^{F,a}	450.11 ± 9.70 ^{D,b}	363.68 ± 6.40 ^{E,c}	402.25 ± 7.47 ^{E,a}	346.98 ± 9.36 ^{D,b}	285.42 ± 4.86 ^{F,c}
		MAP	627.44 ± 9.10 ^{E,a}	453.65 ± 9.48 ^{D,b}	371.68 ± 3.45 ^{E,c}	414.78 ± 12.85 ^{E,a}	355.10 ± 14.39 ^{CD,b}	300.44 ± 9.21 ^{E,c}
12.5 (T)	4	Air	751.10 ± 23.62 ^{B,a}	548.29 ± 1.84 ^{BC,b}	487.89 ± 4.71 ^{B,c}	536.46 ± 9.30 ^{A,a}	471.76 ± 5.67 ^{A,b}	383.75 ± 10.95 ^{B,c}
		MAP	834.45 ± 16.43 ^{A,a}	624.84 ± 17.98 ^{A,b}	498.02 ± 4.72 ^{A,c}	539.51 ± 14.49 ^{A,a}	475.94 ± 19.75 ^{A,b}	413.25 ± 5.77 ^{A,c}
	20	Air	707.00 ± 6.44 ^{C,a}	550.97 ± 13.18 ^{B,b}	411.63 ± 4.45 ^{C,c}	476.59 ± 16.98 ^{B,a}	417.20 ± 9.55 ^{B,b}	279.44 ± 10.08 ^{F,c}
		MAP	724.05 ± 8.54 ^{B,a}	604.09 ± 12.61 ^{A,b}	431.18 ± 17.75 ^{C,c}	476.66 ± 5.23 ^{B,a}	427.51 ± 9.77 ^{B,b}	331.16 ± 1.07 ^{C,c}

Values are presented as average ± standard deviation (SD) (n = 3). $^{A-F}$ Different superscripts uppercase letters are significantly different within columns (Duncan's MRT, $p \leq 0.05$). $^{a-c.}$ Different superscripts lowercase letters are significantly different within rows (Duncan's MRT, $p \leq 0.05$).

Table S3. Effect of temperature (4 °C and 20 °C) and packaging atmosphere (Air and MAP) during storage on color parameters (Chroma* and Hue*) of *Salicornia europaea* shoots with different salinity levels (C and T).

Salinity level (g/L NaCl)	Temperature (°C)	Atmosphere	Chroma*			Hue*		
			Time (h)			Time (h)		
			48	96	120	48	96	120
0 (C)	4	Air	20.05 ± 1.27 ^{C,c}	22.15 ± 0.20 ^{B,b}	23.17 ± 0.77 ^{AB,a}	−52.21 ± 0.82 ^{B,b}	−55.39 ± 0.30 ^{BC,a}	−54.57 ± 1.69 ^{BC,a}
		MAP	21.95 ± 0.12 ^{ABC,b}	26.23 ± 0.21 ^{A,a}	17.72 ± 1.34 ^{CD,c}	−54.28 ± 1.42 ^{AB,b}	−57.01 ± 0.09 ^{ABC,a}	−51.88 ± 0.15 ^{C,c}
	20	Air	20.74 ± 1.52 ^{BCb}	22.54 ± 0.09 ^{B,a}	20.01 ± 1.88 ^{BC,b}	−52.32 ± 1.08 ^{B,c}	−57.10 ± 0.24 ^{ABC,b}	−59.97 ± 1.90 ^{A,a}
		MAP	24.64 ± 0.27 ^{AB,a}	25.90 ± 0.26 ^{A,b}	13.66 ± 1.41 ^{D,c}	−55.17 ± 0.81 ^{AB,b}	−59.86 ± 0.02 ^{A,a}	−56.62 ± 1.70 ^{AB,b}
12.5 (T)	4	Air	22.91 ± 1.27 ^{ABC,a}	23.18 ± 1.66 ^{AB,a}	24.77 ± 0.74 ^{A,a}	−52.66 ± 1.70 ^{B,b}	−54.79 ± 0.96 ^{C,ab}	−56.12 ± 0.74 ^{ABC,a}
		MAP	25.92 ± 0.28 ^{A,a}	21.55 ± 1.20 ^{B,b}	16.93 ± 1.23 ^{CD,c}	−57.39 ± 0.18 ^{A,a}	−56.78 ± 1.10 ^{BC,ab}	−53.73 ± 1.85 ^{BC,b}
	20	Air	24.47 ± 1.61 ^{AB,a}	23.83 ± 0.60 ^{AB,a}	17.42 ± 1.81 ^{CD,b}	−54.36 ± 0.60 ^{AB,b}	−58.37 ± 1.48 ^{AB,a}	−56.86 ± 0.61 ^{AB,a}
		MAP	23.50 ± 1.48 ^{ABC,ab}	24.21 ± 1.37 ^{AB,a}	22.41 ± 0.97 ^{AB,b}	−52.84 ± 0.67 ^{B,c}	−56.23 ± 1.15 ^{BC,b}	−59.24 ± 0.35 ^{A,a}

Values are presented as average ± standard deviation (SD) (n = 3). $^{A-D.}$ Different superscripts uppercase letters are significantly different within columns (Duncan's MRT, $p \leq 0.05$). $^{a-c.}$ Different superscripts lowercase letters are significantly different within rows (Duncan's MRT, $p \leq 0.05$).

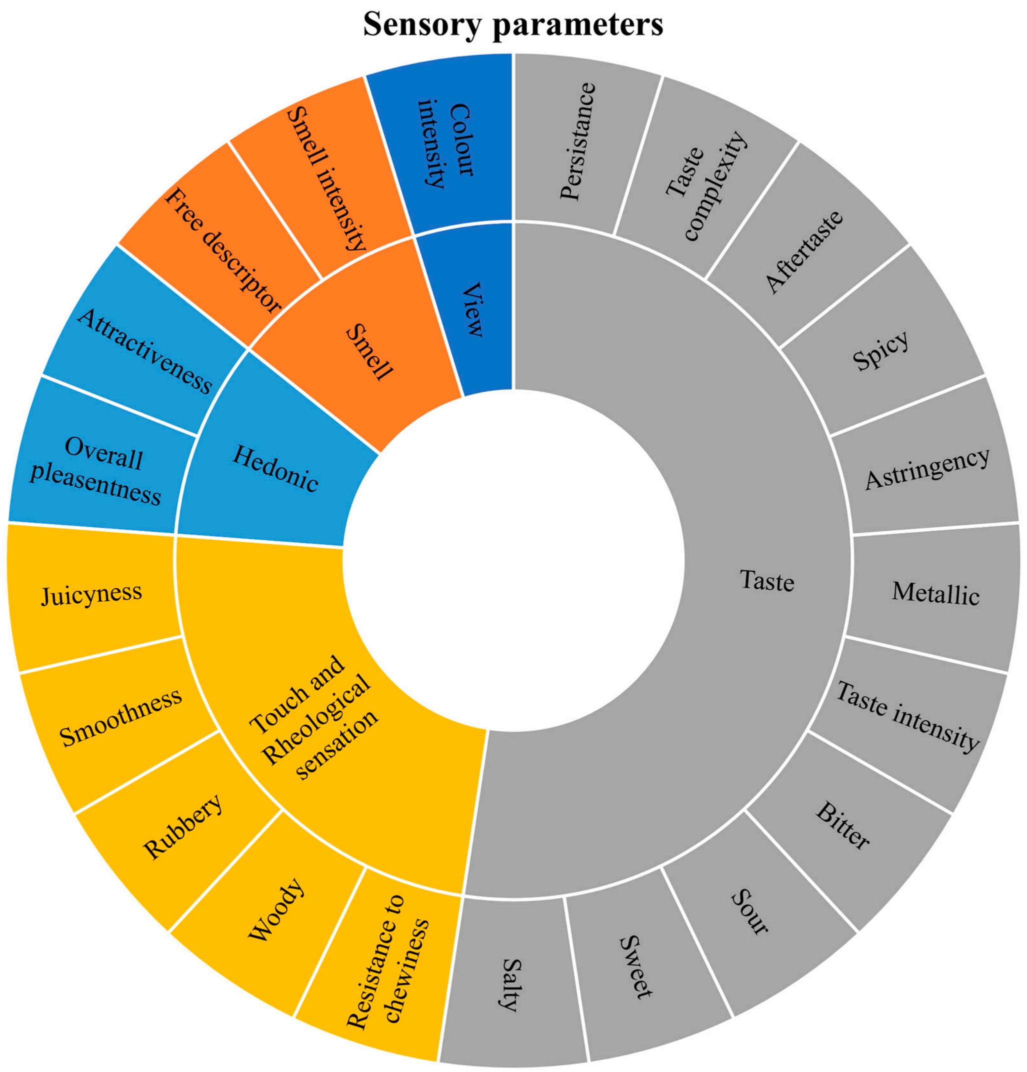

Figure S1. Sensory parameters selected and evaluated at harvest in *Salicornia europaea* shoots grown in hydroponic systems with different salinity levels (C and T).

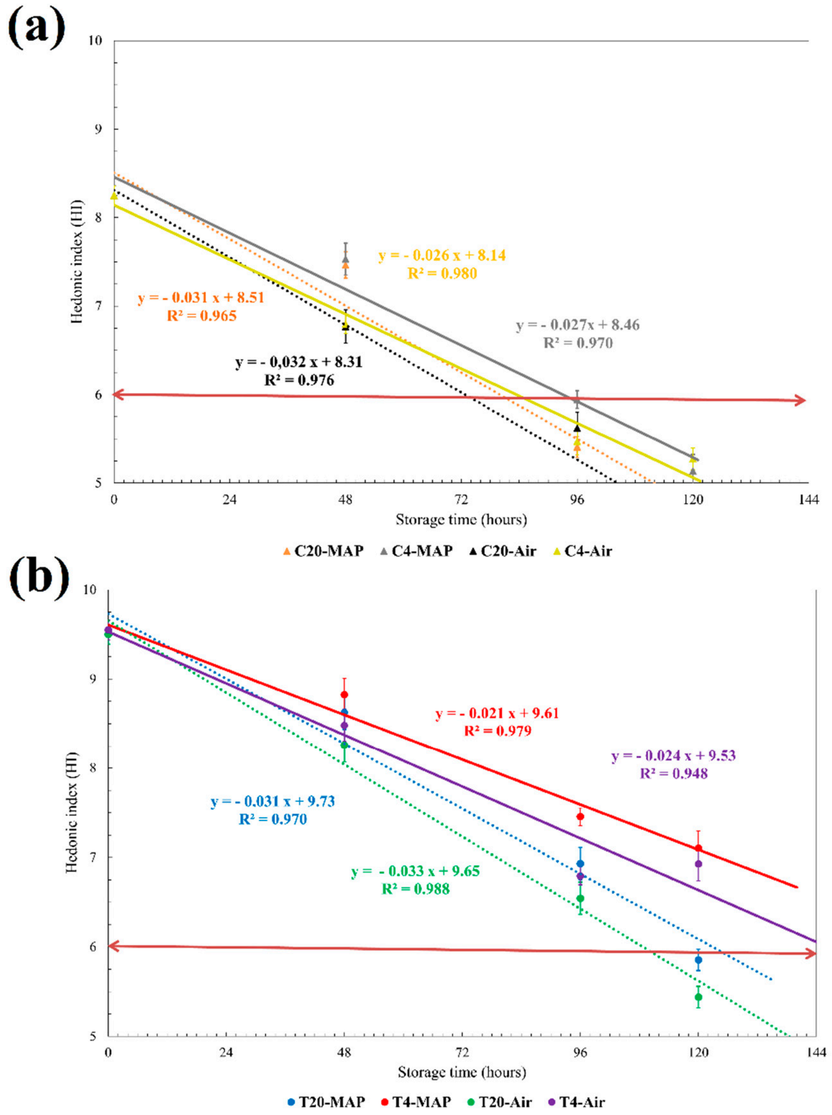

Figure S2. Line regression of the trend of hedonic index (HI) during storage at different temperatures (4 °C and 20 °C) and packaging atmospheres (Air and MAP) of *Salicornia europaea* shoots grown in hydroponic systems with different salinity levels: (**a**) C (0 g/L NaCl); (**b**) T (12.5 g/L NaCl).Values are the average (bars indicate ± SD) (n = 3).
